# Traditional and new lifestyle interventions to prevent breast cancer recurrence (TANICA): a qualitative study

**DOI:** 10.1007/s00520-023-07663-0

**Published:** 2023-03-17

**Authors:** Tanisha F. Aflague, Monica K. Esquivel, Kristi Hammond, Bernice Delos Reyes, Joseph Keawe‘aimoku Kaholokula

**Affiliations:** 1 University of Guam, College of Natural and Applied Sciences, Mangilao, Guam, USA; 2 University of Hawai‘i, College of Tropical Agriculture and Human Resources, Honolulu, Hawai‘i USA; 3 University of Hawai‘i, John A Burns School of Medicine, Honolulu, Hawai‘i USA

**Keywords:** Breast cancer, Native Hawaiian, Pacific Islanders, Asians, Obesity

## Abstract

**Purpose:**

Breast cancer is the second cause of death from cancer in Guam and Hawai‘i and disproportionately impacts Native Hawaiian, CHamoru, and Filipino women. Although a few culturally informed interventions addressing breast cancer survivorship exist, none have been developed or tested for Native Hawaiian, CHamoru, and Filipino women. To address this, the TANICA study began with key informant interviews in 2021.

**Methods:**

Purposive sampling and grounded theory approaches were used to conduct semi-structured interviews with individuals experienced in providing healthcare or implementing community programs and/or research with ethnic groups of interest in Guam and Hawai‘i. A literature review and expert consultation identified intervention components, engagement strategies, and settings. Interview questions aimed to understand the relevance of evidence-based interventions and explored socio-cultural factors. Participants completed demographics and cultural affiliation surveys. Interviews were independently analyzed by trained researchers. Themes were mutually agreed upon by reviewers and key themes were identified based on frequencies.

**Results:**

Nineteen interviews were conducted in Hawai‘i (*n*=9) and Guam (*n*=10). Interviews confirmed the relevance of most of the previously identified evidence-based intervention components for Native Hawaiian, CHamoru, and Filipino breast cancer survivors. Ideas around culturally responsive intervention components and strategies emerged that were shared across and unique to each ethnic group and site.

**Conclusion:**

Evidence-based intervention components appear relevant, yet cultural and place-based strategies are needed for Native Hawaiian, CHamoru, and Filipino women in Guam and Hawai‘i. Future research should triangulate these findings with the lived experiences of Native Hawaiian, CHamoru, and Filipino breast cancer survivors to develop culturally informed interventions.

**Supplementary Information:**

The online version contains supplementary material available at 10.1007/s00520-023-07663-0.

## Introduction

Cancer is the second leading cause of death in nearly all the US Affiliated Pacific Islands (USAPI) and Hawai‘i. Breast cancer, specifically, is the second highest contributor to cancer mortality in Guam and Hawai‘i [1, 2]. Women with obesity who are diagnosed with breast cancer have an increased relative risk for recurrence (40 to 50%) and mortality (53 to 60%) [3, 4]. Native Hawaiian and CHamoru (indigenous people of the Mariana Islands including Guam) women have higher rates of obesity than their non-Hispanic White and Filipino counterparts [5]. Filipinos, a large ethnic group in Hawai‘i and Guam, also experience a high rate of obesity-related chronic diseases, including cancer [6]. Obesity is a modifiable risk factor amenable to lifestyle interventions.

Research on cancer survivorship in other populations has linked better survival after breast cancer treatment among women with a healthy body weight and lifestyle behaviors, such as being physically active and eating a high fiber diet and less saturated fat [3]. Evidence suggests that lifestyle interventions leading to weight loss among women with excess body weight can reduce breast cancer recurrence [4, 7]. Studies of lifestyle interventions among Pacific Islanders have been found to achieve modest weight loss and little is known about the maintenance of weight loss over time [8]. Traditional lifestyle interventions may be less effective for certain ethnic populations due to socioeconomic and sociocultural factors that hinder successful weight loss management, which are not accounted for by these interventions [9].

Indigenous and traditional diet patterns among Pacific Islanders and Filipinos were rich in tropical fruits and vegetables that are good sources of cancer preventative nutrients, such as fiber and antioxidants, and are low in saturated fat [10]. Gathering and growing tropical produce, hunting, and fishing was part of the daily physical activity routine for Pacific Islanders and Filipinos. Over the last century these populations rapidly transitioned to a Western diet and lifestyle consisting of imported and highly processed, calorie-dense foods and more sedentary behaviors [11, 12]. Native Hawaiians, CHamorus, and Filipinos share similar histories of colonization and migration (Filipinos) and shifts from subsistence to cash economies in their island homes, thus altering their food ecosystem and creating an obesogenic environment.

Prior studies have elucidated the importance of food, family, and cultural celebration in Pacific Islander and Filipino communities and how they may influence food choices and lifestyle behaviors, which despite changes to traditional physical activity and diet, remain unchanged. Ancestral crops (*taro*) in Hawai‘i connect people to the land through an origin story or common belief as stewards of the land [13]. In Guam, traditional celebrations among community or family members called *fiestas*, involve gathering to prepare an abundance of food and partygoers may consume up to 3,858 kcal/meal [14]. Like the CHamoru diet, rice is a staple and the center of Filipino culture and celebrations such as a rice festival, a dedicated day to acknowledge the rice harvest in the Philippines. Filipino-Americans continue to include rice as a major part of their diet [15].

Previous research has also identified ideal settings for cancer prevention and lifestyle intervention for Pacific Islanders, such as churches or faith-based locations, workplaces, and sites within communities [16–19]. Using cultural context to change health behavior patterns can promote healthier lifestyles while preserving the cultural traditions and values of Pacific Islanders and Filipinos as their social, political, and economic environments evolve [20]. Engaging communities in Guam and Hawai‘i to identify the unique sociocultural factors that contribute to weight loss maintenance among Pacific Islander breast cancer survivors is needed to effectively address the operative social determinants of health and culturally acceptable, place-based interventions for survivorship.

To improve the effectiveness and cultural responsiveness of lifestyle interventions for Pacific Islander and Filipino breast cancer survivors, the Traditional and New Lifestyle Interventions to Prevent Breast Cancer Recurrence (TANICA) study was initiated in 2021. TANICA was carried out in two phases by Cassel et al. [21]. Phase I was designed to engage community collaborators to learn more about their experiences and interactions with breast cancer survivors and perspectives on changing lifestyle behaviors considering the multi-level influences and the ideal settings to promote and support behavior change. Phase II was to develop an intervention framework specific to Native Hawaiian, CHamoru, and Filipino breast cancer survivors in Guam and Hawai‘i.

This paper will report on the findings from Phase I of TANICA with the following specific aims: 1) identify culturally acceptable ways of engaging women breast cancer survivors, 2) explore the relevance of intervention components from evidenced-based strategies for lifestyle interventions for achieving weight loss among breast cancer survivors who are overweight or obese, 3) determine ideal intervention settings across multiple domains of influence, and 4) explore cultural values that support intervention activities.

## Methods

### Research design and framework

A qualitative study design that included interviews was used. A purposive sampling approach was used to recruit participants and obtained data underwent thematic analysis. To examine the multiple levels that influence obesity prevention through diet and health promotion [20, 22], the Social Ecological Model (SEM) [23] was used to guide recruitment and interview questions to consider the complex interplay between an individual and their environment from social to physical and at the macro-level (e.g., stigmas, socioeconomics, political). For this study, we will represent the different levels of influence across the SEM as individual (e.g., personal history or knowledge of breast cancer), interpersonal (e.g., family support, social network), organizational (e.g., healthcare service, worksite support, safe spaces for walking), community (e.g., access, design, relationships between organizations, built environment), and policy (e.g., national, state, local and organizational policies and laws) with sociocultural (e.g., traditional practices, stigmas) that intersects all levels.

### Participant recruitment

The recruitment of participants targeted individuals who have experience in providing health care, implementing community health promotion programs, engaging in advocacy, and/or conducting health-related research with the communities of interest in Guam and Hawai‘i. Existing community (i.e., American Cancer Society, support groups, health care professionals) and professional (e.g., Cancer Research Centers) networks that engaged with breast cancer survivors were used to generate a list of potential participants. They were categorized to represent an SEM level - individual, interpersonal, organizational, community, and policy—from each of the respective research sites. For example, a person who worked with CHamoru breast cancer survivors on an individual level was categorized as operating at the individual level of the SEM, while a person who worked on policies or public health programs focused on Native Hawaiian breast cancer survivors was categorized as operating at the policy level.

The potential participants were contacted by phone or email to assess interest and availability, then to screen further for eligibility. In addition to having experience in working with the communities of interest across different SEM levels, the participants also needed to be 18 years or older and English speaking. To ensure we gathered a broad range of perspectives, the original recruitment goal called for one to two representatives for each of the five levels of the SEM within the Hawai‘i (*n*=5–10) and Guam (*n*=5–10) sites, separately, for a total of 10–20 participants. Recruitment goals were met in which 9 and 10 key informants in Hawai‘i and Guam, respectively, were interviewed. Saturation (i.e., no new concepts were elicited) was reached by the 9^th^ and 10^th^ participants at Hawai‘i and Guam, respectively [24].

### Procedures

#### Interview question development

Interview questions were developed to guide the discussion, guided by a review of existing literature of lifestyle interventions for breast cancer survivors and for Native Hawaiian, CHamoru, and Filipino as well as other methods (i.e., existing knowledge of and consultation with experts that work with Native Hawaiian, CHamoru, and Filipino). Specifically, the literature review identified appropriate intervention components, engagement strategies and settings. Lifestyle behaviors protective against breast cancer recurrence that were identified included approximately 150 min/week to include aerobic and/or resistance exercises with a trainer, calorie restriction, utilization of tools and technology for self-monitoring, increased fiber intake, increased fruit and vegetable intake, and/or specific diets (i.e. low-carb, low-fat) [25]. Engagement methods and strategies included mobile health, phone calls, e-mail, mailing, and digital technology [25]. Implementation settings included health and fitness facilities, community centers, and in online formats [25]. Interview questions are shown in the Supplementary Information 1 (Supplementary Information_1.pdf).

#### Data collection

After participants consented, they were asked to self-report demographic (i.e., age, sex, and ethnicity), breast cancer history, and cultural affiliation. Data collection occurred during the COVID-19 pandemic when mask-wearing and social distancing restrictions were in place. Participants were provided the option to complete interviews in person or online video conferencing (i.e., Zoom). For online interviews, research staff provided the link via email to a private, password-required video conferencing room. In-person interviews were conducted at a location most convenient for the participant and in an area where a private conversation (e.g., research office space, participant’s workplace, or coffee shop) could take place. Local safety protocols were followed wherever interviews took place. Interviews were audio recorded, transcribed verbatim by research assistants, and uploaded into ATLAS.ti software for thematic analysis.

### Measures

Participants reported their ethnocultural affiliation with their (race/ethnicity) using a four-item cultural affiliation questionnaire which assessed: Degree of affiliation with their cultural group, feelings toward their cultural group, knowledge about their cultural group, and the impact their cultural group had on their own lifestyle practices [26]. The responses range from 1 (very knowledgeable, very positive, or very involved) to 5 (not knowledgeable at all or very negative). Scores range from 4 to 20 with lower scores indicating a stronger cultural identity. Past studies have found correlations between this sub-scale and other health outcomes such as hypertension among Native Hawaiians [26, 27]. Participants were also asked to identify the levels of the SEM they felt they represented or operated within.

Key informants were asked a series of questions organized around two areas: 1) sociocultural and 2) intervention components and settings. For the sociocultural area, they were asked to describe their culture and cultural values. These questions were meant to establish similarities and differences between culture and cultural values of the key informants and the populations of interest but also helped to establish a mutual understanding of the context of responses to subsequent questions. Next they were asked their concept of health and to what extent physical activity and diet influence health. Evidence-based intervention components, engagement approaches, and settings identified from the literature review were included in the question set for the second topic area and discussed during the interview using question probes. Key informant interview questions are available in the online supplement.

### Data analysis

Demographic variables were analyzed for descriptive purposes (i.e., mean and standard deviation for continuous variables and frequency and percentage for categorical variables). Cultural affiliation scores were calculated using the sum of all four (4) subscale question responses and presented as means and standard deviation (SD) for each site. Scores could range from 4 to 20 with a lower total score indicating stronger cultural affiliation to the individual’s (race/ethnic) group. Participants’ self-identified SEM levels were tallied for comparison with the researchers’ identified levels to determine congruence and representation of all SEM levels in the results.

Transcripts from interviews were uploaded into ATLAS.ti Windows (Version 22.0.6.0) with identifying information redacted before analysis. Trained researchers (two from each location) used a grounded theory approach to conduct the initial and intermediate coding, applying constant comparative analysis. Each interview was reviewed by two independent researchers to identify themes and code transcripts. Reviewers created codes based on the research aims: 1) culturally acceptable ways of engaging women breast cancer survivors, 2) intervention implementation strategies and components, 3) intervention settings, and 4) cultural values that support intervention activities. Researchers met to determine consensus and if there were discrepancies, consensus was obtained. The most frequently mentioned themes were reviewed quantitatively. Key themes were determined if the theme was mentioned by a minimum of 50% of participants in a geographic location.

## Results

### Participant characteristics

The characteristics of the participants interviewed are summarized in Table 1. Most participants were female (*n*=17) and were of Filipino, Native Hawaiian, and/or CHamoru descent (*n*=15). There was an agreement between participants and researchers for SEM level representation; however, participants identified more than one level of the SEM in which they operated, whereas the researchers assigned participants to just one level each.

**Table 1:** Key informant interview participant characteristics

	Hawai‘i (*n*=9)		Guam (*n*=10)		Total (*n*=19)	
**Sex**	***n*** **(%)**					
Female	9 (100)		8 (80)		17 (89.5)	
Male	0 (0)		2 (20)		2 (10.5)	
**Age group**	***n*** **(%)**					
30–39 years	0 (0)		2 (20)		2 (10.5)	
40–49 years	1 (11.1)		4 (40)		5 (26.3)	
50 years and older	8 (88.9)		4 (40)		12 (63.2)	
**Race/ethnicity**^1^	***n*** **(%)**					
CHamoru	0 (0)		5 (50)		5 (26.3)	
Filipino	3 (33.3)		4 (40)		7 (36.8)	
Native Hawaiian	3 (33.3)		0 (0)		3 (15.8)	
Caucasian	4 (44.4)		0 (0)		4 (21.0)	
Asian	5 (55.6)		2 (20)		7 (36.8)	
Hispanic	1 (11.1)		0 (0)		1 (5.3)	
Palauan	0 (0)		1 (10)		1 (5.3)	
Mayan	0 (0)		1 (10)		1 (5.3)	
More than 1 Race/Ethnicity	4 (44.4)^1^		2 (20)^1^		6 (31.6)	
**Cultural affiliation**	Mean ± SD					
Mean score ± SD	7.0 ± 3.0		7.0 ± 4.4		7.0 ± 3.86	
**SEM level representing**^**2**^	Self Identified	Researcher Identified	Self Identified	Researcher Identified	Self Identified	Researcher Identified
	**n**					
Individual	4	2	5	2	9	4
Interpersonal	2	2	6	2	8	4
Organizational	2	2	6	2	8	4
Community	3	2	6	2	9	4
Policy	4	1	6	2	10	3

^1^Participants selected all race/ethnic groups that applied, counts represent all ethnic groups selected by participants

^2^Participants were able to select more than one social ecological model (SEM) sector

### Emergent themes from interviews

The themes that emerged from key informant interviews were consistent with the review of literature. These themes were organized by 1) intervention components, 2) intervention strategies, 3) implementation venues, and 4) cultural values.

#### Intervention components

Key informants recommended physical activity, nutrition/diet, and other intervention components (Table 2). For each intervention component cultural considerations were noted. Participants from Hawai‘i and Guam highlighted the need for family involvement in the food preparation activities as part of nutrition/diet intervention components as exemplified in the following quotes:

Family is a really important thing and getting to be with family and also just having the cooking and preparing stuff and being with family, it means a lot. (Hawai‘i KI#06)

If a female, a mother and a wife, is going to cook for the entire family, right, they’re going to cook what the entire family wants to eat. Or they’re so busy, they just eat whatever’s there or take it on the way home. (Guam KI#10)

**Table 2:** Intervention component themes by study location

	Hawai‘i	Guam	Both
Physical Activity	Culturally tailored Dance^2^ Link with existing community events Fun	Dance (Zumba, hula)^2^ Group activity linked with a community event Outdoor activity Trackers^1^ Safe and fun Involve friends/survivors	Dance^2^ Fun Linked with a community event
Nutrition/Diet	Food preparation Involve family/Feeding the family Reflective of cultural foods/foods familiar to participants	Nutrition education/ Cooking class Involve family/Feeding the family (Healthy) Traditional food preparation Filipinos: Grow food/Garden Prescriptive/Authority^2^	Involve feeding family Food preparation/Cooking Class Traditional foods
Other		Mental health/Stress management	

^1^Evidence-based strategies and components found in the literature review

^2^Common practice or existing knowledge guided by experts

Culturally tailored nutrition information and physical activity were themes among participants working in both Hawai‘i and Guam and represented in selected quotes here:

When it comes to diet, you may just have to consider if there’s cuisine differences based off (of) culture in terms of what ingredients would be readily available in a Filipino home versus what ingredients would be readily available in a CHamoru home. And I think that that’s where the difference would be in terms of what are staples in this home versus what are staples in this home. (Guam KI#5)

Try not to call it exercise because that has a bad connotation or a negative connotation, but just play with the kids, or walk with your friends, do something that’s enjoyable because otherwise, you’re not going to continue it. (Hawai‘i KI#05)

Only participants in Guam referenced stress management as an intervention component, like this quote:

...one thing she always tells me is, some activity or being involved in certain things are not worth it to her and her health because of the stress that it brings, and she tries to avoid stress as much as possible. (Guam KI#8)

#### Intervention strategies

Participants identified key strategies for communicating with intervention participants and offered recommendations for culturally acceptable methods for engagement (Table 3). Email and phone or text were common communication strategies across both Hawai‘i and Guam, where radio/newspaper was unique to Hawai‘i and word of mouth was emphasized in Guam. One participant shared:

if we’re talking about the breast cancer survivors, you’re looking at a demographic of between like 40 and 80. A lot of them don’t really have the digital know-how. Yeah, so with this current pandemic situation, it’s the actual calling that works. So pre-pandemic or post-pandemic, a lot of them still, they love the in-person. They understand it more in-person when you’re able to kind of just talk to them and explain why and what’s going on. (Guam KI#3)

**Table 3:** Intervention implementation strategy themes by study location

	Hawai‘i	Guam	Both
Communication	Email^1^ Social media^1^ Phone^2^ Radio/newspaper Filipinos:Via church	Social media^1^ Phone/text^2^ Apps/Digital technology^1^ Word of mouth/Support groups	Social media^1^ Phone/text^2^
Culturally Acceptable Engagement	Building trust/establishing trust first Filipinos: Authority figure	Creating a sense of community (survivors) Filipinos: Authority figure	Trust Filipinos: Authority figure

^1^Evidence-based strategies and components found in the literature review

^2^Common practice or existing knowledge guided by experts

A key strategy to implementing interventions was building trust and for Filipinos, the theme of an authority figure arose in both locations. A participant from Hawai‘i expressed this regarding trust:

People out here they need to trust any new program because so many things out here, programs come and go, people come and go, people come in with a good idea, they’re going to help all these people and then they’re gone…. they need to trust the people that are leading it or involved so that it’s not just another thing. Another thing that people come out here, they think they’re going to help us, but they’re going to be gone. (Hawai‘i KI#05)

An authority figure was mentioned in both sites only for Filipinos, as a key strategy for implementing the intervention. Participants in Guam echoed the sentiment in Hawai‘i:

authority in the Filipinos, use of authority, so if the pastor says, or the doctor says, then they might be more willing. I don’t think Hawaiians and authority go together. I don’t think it makes a difference for them. (Hawai‘i KI#04)

Filipinos like directives. Filipino women, you’re the authority, you’re the dietitian [and they would say] ‘Well, my dietitian, she told me this. This is what she said.’ And they’ll comply. Filipinos are more compliant. (Guam KI#1)

#### Implementation venues

Several implementation venues were identified by the key informants (Table 4). Many of these were supported by the literature, such as existing community settings most frequently reported across both sites as a viable venue for intervention implementation. Churches were another venue mentioned, yet only emphasized in Hawai‘i. Notably, a mix of in-person and virtual venues were also shared for both nutrition and physical activity intervention components. In Hawai‘i, convenient locations were emphasized:

First of all, have it at a time that they can actually go. If they’re working women, then find a way to address it so they can do it on work time. (Hawai‘i KI#05)

**Table 4:** Intervention implementation strategies by setting and study location

	Hawai‘i	Guam	Both
Settings- Physical Activity	Church/Faith-based settings^2^ Home- pre-recorded videos Gym Community environments if safe/walkable^2^	Community center^1^ Indoor space Outdoor park/beach Home/Community garden^2^ Need safe walkable spaces^2^	Community settings^1^ Safe/walkable spaces
Settings- Nutrition/Diet	Convenient and familiar to participants Filipinos: Churches^2^ Community centers^1^	Education kitchens Participants’ homes Apps/Online^1^	Existing organizations

^1^Evidence-based strategies and components found in the literature review

^2^Common practice or existing knowledge guided by experts

In Guam, recognizing the outdoor temperatures came up when deciding on a venue:

In Guam, it is hot. There are gyms. There is a swimming pool. There’s beaches. In terms of walking or aerobic exercise, just really finding those perfect times to do it [outdoors]. In the morning is really great or later in the afternoon, because it is really hot when it’s like 12 to 3. (Guam KI#6)

#### Cultural values

Themes around key cultural values to consider when delivering the interventions emerged from key informant interviews. Similar across both sites are family, food preferences, and a propensity for group/community needs over individual needs (Table 5). Participants in both sites shared:

Family. We’re very collectivistic, so we take care of each other. We think of the greater good for all instead of the individual. Respect our elders, take care of our elders. (Hawai‘i KI#08)

it’s harder to be successful when your unit is not. And so I think the whole family unit needs to be involved in. (Guam KI#10)

**Table 5:** Emerging themes of key cultural values for intervention design and implementation by study site

	Hawai‘i	Guam	Both
Key Cultural Values	Family Cultural food preferences Privacy (Filipino) Faith/spirituality Group vs. individual	Family Cultural food preferences Respect Sense of community	Family Cultural food preferences Group (community) vs. individual

Specific to Guam, the value of respect surfaced with one participant saying:

a value that’s really important to me is called *finet taotao*, which means you treat others with absolute respect. And it’s not a respect that’s earned, it’s respect just by virtue of them being members of humanity. (Guam KI#2)

In Hawaii, the Filipino community’s desire for privacy also emerged:

keep it to themselves, not expose their struggles or share their struggles with people outside their family. I think even with their own family, they don’t want to share how hard treatment was. (Hawai‘i KI#08)

#### Themes across the social ecological model

**Fig. 1:**
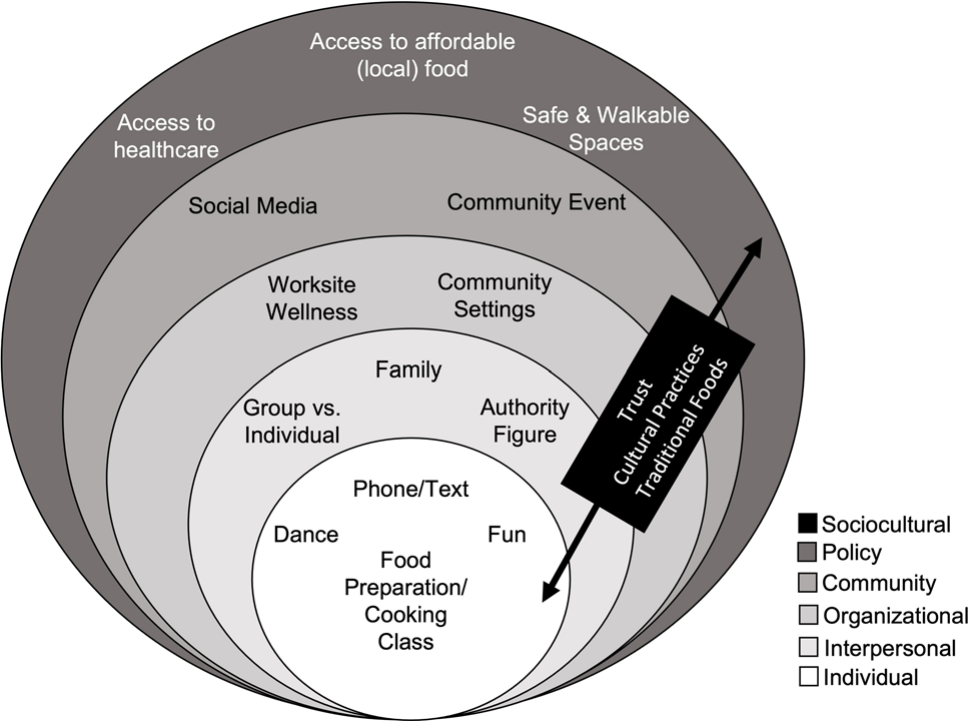
Emerging themes of lifestyle intervention implementation activities and components for breast cancer survivors from key informant interviews in both Guam and Hawai‘i by Social Ecological Model (SEM) level

Determinants at all levels of the SEM were perceived as relevant for an intervention program for female breast cancer survivors in Guam and Hawai‘i. Figure 1 depicts the SEM populated withthe key informant interview themes identified in this research. Themes were elucidated across domains for intervention components and implementation strategies at the individual (e.g., dance and food preparation activities), interpersonal (e.g., family support, social network), organizational (e.g., healthy food/drink options/policy at the workplace or at church, physical activity fit into work schedule or at church), community (e.g., social media and community-based programs/events), and policy (e.g., policy to support the built environment that promotes safe and walkable spaces, healthcare policy reform to improve access to nutrition education/counseling, local food cost/procurement policy) levels and the cross-cutting sociocultural (e.g., establish trust, incorporate cultural practices and traditional foods) factors.

## Discussion

Most intervention strategies reviewed were found suitable for Native Hawaiian, CHamoru, and Filipino female breast cancer survivors, such as, increasing physical activity in small groups at a community-based setting, increasing (local) fruits and vegetables with nutrition education, cooking class, and/or coach (es) (e.g., authority figure) and using tools to track progress (e.g., pedometer, fitness/diet Apps) at the individual and interpersonal levels. However, cultural- and geographic-specific strategies were identified that could enhance the acceptability, effectiveness, and sustainability of these interventions.

Culturally specific themes were found throughout the SEM in this study, which are absent in most lifestyle interventions. At the individual level, participants in this study highlighted the need for cultural foods and traditional recipes/methods to be included as part of the education. The family and social network of Native Hawaiian, CHamoru, and Filipino breast cancer survivors should be included or involved in the intervention activities at the interpersonal level.

For each ethnic group, the support person or group to be included or involved in the intervention program were different. Key informants explained that members of the family and extended family (e.g., social network) have different roles when supporting survivors. Cross-cutting individual, interpersonal, organizational, and community levels is the intervention implementation strategy theme of “creating a sense of community” in all aspects of the intervention for survivors, physical activity trainers, dietitians/nutrition educators, family, and extended family and is critical for CHamorus and Filipinos alike in Guam. A sociocultural level theme is creating a community in that builds trust. A complementary intervention implementation strategy that potentially creates a (virtual) community in an intervention is the use of social media, which uses contemporary practices to operationalize a traditional practice. Leveraging existing community events, activities, and settings are intervention components that can be used to reinforce the sense of community.

The workplace was identified in Guam as a setting that will support the intervention to influence healthy options or healthy eating policies that is captured in the following quote:

…on an organizational level dealing with—they come back to work, there’s your donuts in the morning, your diet sodas and whatever, your sodas...so I think in that type of setting, it’s very difficult. (Guam KI#1)

Breast cancer survivors’ work schedules were identified as a consideration when designing the physical activity component as women are eager to return to work after treatment. Worksite wellness policies that allow paid time off for disease prevention activities (e.g., annual check-ups, physical activity, brown bag sessions) have shown to support long-term lifestyle changes [28]. In Hawai‘i, policy change at the organizational level is another means to support the diet/nutrition intervention components when a participant stated:

…organizational policies right local foods, traditional foods at government sponsored events. Only healthy drinks, coconut water or water at church events, no fruit punch. So those are examples of little p strategies that can be really effective right because you want to make healthier options be the most widely available options. (Hawai‘i KI#08)

Other potential policy changes on a broader scope that surfaced was access to affordable (local) foods in Guam and access to healthcare providers (i.e., registered dietitian nutritionist) in Hawai‘i, elucidated in the following quotes:

If everybody had access to a dietitian, holy moly, they are amazing. Well, you might yeah change reimbursement. Yeah. To get more dietitian, you have to change reimbursement. You have to make it part of the package. It has to be incorporated into value-based payments and payment transformation. Right now it's not. We've been advocating for it. (Hawai‘I KI#07)

It was hard because we like packaged foods. It’s cheaper, right? I mean, when you look at the choices...the cost is so important...because I’ve been to the farmer’s market many times and those vegetables are not cheap. (Guam KI#1)

Consistent with the literature reviewed, intervention implementation strategies that were suggested by the participants were to utilize church and/or faith-based organizations [16]. In Hawai‘i, the use of churches was specific to Filipinos to incorporate cultural practices around their faith and spirituality. Although not a key theme found in Guam, church was mentioned as a setting as part of the community setting and participants spoke of their personal spirituality and/or faith. Christianity and Catholicism were introduced across the Pacific with colonization and many CHamorus and Filipinos practice Catholicism where 85% of the population in Guam are Roman Catholics [29]. Thus, leveraging the church, religious practices, and spirituality/faith are ideal strategies and components for an intervention program in these locations.

Church parishioners or fellows in faith are also well-established social networks. In Guam, an effective communication strategy identified was by “word of mouth” through social networks, like support groups or mentors. Key informants emphasized engagement would be most effective between social networks with shared experiences or interests who have established trust, like survivors and other survivors or among friends or family. Establishing trust and demonstrating respect for all is critical when conducting research or implementing programs in these locations related to the historical trauma (e.g., colonization, war, weapons testing). Community-based participatory research (CBPR) can be used to address these concerns and has shown to be an effective research method in underserved communities and minority populations [30–32]. Community engagement strategies used in CBPR have been through community health workers of the same population as the community to be engaged [33, 34].

Breast cancer is a highly stigmatized disease and survivors are faced with many challenges, physical and psychosocial, further supporting CBPR to shape culturally-grounded programs.

Addressing stress and mental health arose as an intervention component to consider in Guam. An intervention component that includes an assessment of perceived health-related stigma with a stress management and/or mental health component is warranted [35]. Interviews were conducted peri-pandemic and mental health was at the forefront of community stakeholders’ minds considering the psychiatric emergencies and heightened anxieties brought on by COVID-19 and the pandemic [36]. The effects of the pandemic will likely linger on for years to come making mental health and stress management valid lifestyle factors to consider in any intervention.

Geographic concordance for intervention setting was outdoors, which was unsurprising, considering the tropical climates in both research locations. On a community and policy level, the concern for a lack of walkable spaces and, in Guam, an emphasis on safe spaces were identified. Filipinos in Hawai‘i may be more Westernized than Filipinos in Guam, close in proximity to the Philippines, and may explain why only Filipinos in Guam would prefer to garden or grow their own food and continue their traditional living practices in the Philippines [2]

Previous research has informed the work of the TANICA study and will be combined with practice-based evidence from key informants’ professional perspectives and experiences representating SEM sector(s). These perspectives will be used with the personal perspectives and experiences of breast cancer survivors to address the complex interplay of lifestyle factors across SEM levels, which is a strength of this study. The three-points of data provide the foundation to inform CBPR methods in future studies. A limitation of this study is that only English-speaking participants were recruited due to researchers and research staff being proficient in English. However, English is one of the official languages in both locations where Hawaiian and CHamoru were the other languages in Hawai‘i and Guam, respectively.

## Conclusion

Our findings suggest that most evidence-based intervention components may be suitable for designing a lifestyle intervention for Pacific Islander and Filipino breast cancer survivors in Hawai‘i and Guam. Additionally, this research has identified unique and shared intervention components and implementation strategies between the two research sites. Nutrition interventions specifically must incorporate cultural foods, cooking practices, and recipes to enhance relevance for the population of interest and interventions should be implemented within the context of the family and community environments in which breast cancer survivors live. Involving key informants, with professional experience in working with Native Hawaiian, CHamoru, and Filipino communities, in developing interventions should be a priority. Future research should triangulate these findings with the lived experiences of Native Hawaiian, CHamoru, and Filipino breast cancer survivors in Hawai‘i and Guam.

## Supplementary information

Key informant interview questions (PDF 71.7 KB).
