# Supplementary material for: Traditional and new lifestyle interventions to prevent breast cancer recurrence (TANICA): a qualitative study
Source: Support Care Cancer. 2023 Mar 17;31(4):218. doi: 10.1007/s00520-023-07663-0 (PMC10020071; doi:10.1007/s00520-023-07663-0)
Supplement: Supplementary file 1 — Key informant interview questions (PDF 71.7 KB). [file 520_2023_7663_MOESM1_ESM.pdf]

Article title: Traditional and New Lifestyle Interventions to Prevent Breast Cancer Recurrence (TANICA): A qualitative study.
Journal name: Supportive Care in Cancer
Author names and affiliation: corresponding author.
Tanisha F. Aflague, PhD, RDN University of Guam, College of Natural and Applied Sciences, Mangilao, Guam, USA; taflague@triton.uog.edu; (corresponding author)
Monica K. Esquivel, PhD, RDN
Kristi Hammond, MS
Bernice Delos Reyes
Joseph Keawe‘aimoku Kaholokula, PhD

Online Resource 1. Key informant interview questions for the Traditional and New Lifestyle Interventions to Prevent Breast Cancer Recurrence (TANICA) study.

Topic	Question
Sociocultural	What is the culture you identify with most?<br><br>What are important values in your culture?<br><br>As a person from outside the [Native Hawaiian/CHamoru/Filipino] community, what is important for me to know about working with breast cancer survivors from your community?<br><br>What do you think a healthy lifestyle is?<br><br>What do you think about the health of breast cancer survivors from your community?<br><br>How much do you believe that what you eat and how much physical activity you do are important components to health?<br><br>Many factors influence a person’s health, one model is the social ecological model (image shown to interviewee). This model identifies individual, interpersonal, community, organizational, and policy factors that influence health. Thinking of yourself and your work in the breast cancer community, which level do you most represent? Why this/these levels?
Intervention components and settings	Having too much of certain types of body fat is a risk factor for breast cancer recurrence. Healthy eating and physical activity can help to reduce excess amounts of this body fat.<br><br>Research among other breast cancer survivors have found that some interventions are helpful in changing eating and physical activity habits that make people more healthy include:<br>● moderate exercise of about 150 minutes/week, aerobic exercise;<br>● calorie restriction with the support of a Registered Dietitian or using of prepackaged meals (i.e., shakes);<br>● developing support systems with technology (mobile applications, pedometers/tracking);<br>● combinations of resistance and aerobic exercise under the supervision of an exercise professional;<br>● Mediterranean diets;<br>● increasing fruit and vegetable intake for a high fiber diet; and<br>● increasing intake of soy foods after, low carbohydrate and low-fat diets.<br>From the list of intervention components found in the literature (mentioned earlier), what are some activities that you think would help the [Native Hawaiian/CHamoru/Filipino] community of breast cancer survivors in [Guam/ Hawai‘i]?

	Other communities have found that mobile health, phone, emails, mailing, digital technology coupled with in-person activities, including multiple forms of accountability, workplace settings, churches or faith-based settings, settings outside the home, other community based-settings and health and fitness facilities are good for providing these cancer prevention and lifestyle interventions.<br>a. Where do you think would be a good place to reach [Native Hawaiian/CHamoru/Filipino]<br>b. breast cancer survivors regarding eating and physical activity?<br>c. What approach/method would be ideal to communicate with [Native Hawaiian/CHamoru/Filipino] breast cancer survivors?<br>i. Which of these approaches would be useful for accountability (if any)?<br><br>What are some characteristics of the [Native Hawaiian/CHamoru/Filipino] community of breast cancer survivors that might facilitate or hinder an intervention addressing breast cancer survivorship?<br><br>What are some characteristics of the [Native Hawaiian/CHamoru/Filipino] community of breast cancer survivors that might facilitate or hinder an intervention addressing excessive body fat or weight loss?<br><br>If there was a new program to address healthy living to promote breast cancer survivorship, how could we make it attractive to [Native Hawaiian/CHamoru/Filipino] females in [Guam/ Hawai‘i]?<br><br>Should lifestyle interventions (to lose weight?) be different for [Native Hawaiian/CHamoru/Filipino] breast cancer survivors?<br><br>In a talk story session (or focus group) with breast cancer survivors, who would you recommend to conduct or facilitate the session? Would you be willing to conduct or facilitate a session?
